# Availability of basic infection control items and personal protection equipment in 7948 health facilities in eight low- and middle-income countries: Evidence from national health system surveys

**DOI:** 10.7189/jogh.14.04042

**Published:** 2024-03-01

**Authors:** Shariful Hakim, Muhammad Abdul Baker Chowdhury, Md Jamal Uddin, Hannah H Leslie

**Affiliations:** 1 Department of Statistics, Shahjalal University of Science & Technology, Sylhet, Bangladesh; 2 Chander Hat Degree College, Nilphamari, Bangladesh; 3 Department of Neurosurgery, College of Medicine, University of Florida, Gainesville, Florida, USA; 4 Department of General Educational and Development, Daffodil International University, Dhaka, Bangladesh; 5 Division of Prevention Science, Department of Medicine, University of California San Francisco (UCSF), San Francisco, California, USA

## Abstract

**Background:**

Hundreds of millions of people become infected globally every year while seeking care in health facilities that lack basic needs like infection control measures and personal protective equipment (PPE). We aimed to evaluate the availability of infection control items and PPE in eight low- and middle-income countries and identify disparities in the availability of those items.

**Methods:**

In this study, we combined publicly available nationally representative cross-sectional health system surveys (Service Provision Assessments by the Demographic and Health Survey Programme) conducted in eight countries between 2013 and 2018: Afghanistan, Bangladesh, the Democratic Republic of the Congo, Haiti, Malawi, Nepal, Senegal, and Tanzania. The availability of infection control items was evaluated using a list of six items (a waste receptacle, a sharps container, disinfectant, single-use disposable or auto-disposable syringes, soap and running water, or an alcohol-based hand rub, and guidelines for standard precautions). PPE includes four items: gloves, medical masks, gowns, and eye protection. We considered these items available in a facility if they were observed in general outpatient areas or any service-specific area (i.e. delivery room).

**Results:**

We analysed data from 7948 health facilities (694 hospitals and 7254 health centres/clinics). Overall, among the infection control items and PPE, most surveyed facilities had high availability of single-use disposable or auto-disposable syringes (91.40%) and latex gloves (92.56%). Of infection control measures, guidelines for infection control were the least available during the survey, with the lowest (6.15%) in Nepal and the highest (68.18%) in Malawi. Of the PPE items, eye protection was the least available during the survey, with the lowest (5.4% in Senegal) and the highest (28.17%) in Haiti. Only 1567 (19.71%) facilities looked to have all the basic infection control materials, and 1023 (12.87%) of the analysed facilities possessed all of the PPE. Within the same country, the availability of items varied more between hospitals and health centres/clinics than between them.

**Conclusions:**

All eight of our study countries experience shortages of the most fundamental standard precaution items to avert infection. Steps must be taken in each of these countries to reduce inadequacies and disparities and enhance efficiency in the conversion of health-system inputs into the facility’s availability of standard precaution items for infection control – to curb the risk of infectious disease transmission.

A core principle of health care is that it should inflict no harm on patients [1,2]. Even so, hundreds of millions of people are put at risk of infection globally while seeking care in health facilities that lack basic needs like infection prevention and control (IPC) measures and personal protective equipment (PPE). IPC measures and PPE are critical aspects of all health systems globally, affecting the health and safety of individuals who use health services and the health care workers (HCWs) who provide the service. Lack of PPE and IPC measures in health facilities puts patients at risk of health care-associated infections (HAIs) and contributes to the spread of antibiotic-resistant infections [3].

HAIs, also known as ‘nosocomial’ infections, are by far the most common adverse outcomes in care delivery. Every year, HAIs afflict hundreds of millions of patients around the world, leading to enormous mortality and financial costs for health systems and frequently resulting in the amplification of epidemics [4]. Infection correlated with unclean births is responsible for 11% of maternal mortality and 26% of neonatal deaths; together, these are responsible for more than one million deaths every year [5]. Among patients receiving care in acute-care hospitals, it is anticipated that 7% of individuals in advanced countries and 15% in low- and middle-income countries (LMICs) are likely to develop at least one HAI during their hospitalisation [6]. Moreover, the burden of HAI is notably higher in developing countries, where the chance of suffering from HAI is two to twenty times higher compared to developed countries [7].

The increasing prevalence of antimicrobial resistance demonstrates that, without proper infection control procedures, health care facilities may operate as permanent reservoirs of resistance or enhance transmission of resistant bacteria both within facilities and in the community. The experience from the Ebola and Coronavirus disease 2019 (COVID-19) pandemics indicates how easily health facilities can act as amplifiers of new pathogens and diseases in the community, how important it is to protect HCWs, and how difficult it is for health systems in low-income countries to provide enough protective equipment and infection prevention to keep their HCWs safe. Without the proper protective equipment, about 8% of health workers in Liberia and about 7% in Sierra Leone died from Ebola virus [8].

During the COVID-19 pandemic, HCWs have been highly vulnerable to getting the infection and spreading the disease to their families, colleagues, and other patients [9]. Several prior studies have found gaps in IPC measures. A 2018 study of 129 557 health facilities across 78 LMICs revealed deficiencies: 61% had handwashing soap, 61% had proper infectious waste disposal, 27% had sterilisation equipment, and 26% had standard precaution guidelines [10]. Statistically significant differences in coverage were present between health facilities by facility type, managing authority, and urban-rural setting. A current analysis of 16 456 health facilities from 18 sub-Saharan African countries published by Kanyangarara et al. found that 26% lacked soap and running water or alcohol-based hand rub, and 87% lacked standard precautions for infection prevention [11]. This study also investigated a wide inequality in access to water, sanitation, and hygiene services between urban and rural settings, as well as public and private facilities.

As far as we are aware, no studies have attempted to investigate the full scope of such gaps or how they are distributed. Our aim was to assess the availability of infection control items and PPE in eight LMICs. We sought to identify disparities in the availability of those items between health facilities in urban and rural settings by ownership of the facility and by facility type, utilising data from nationwide representative samples of facilities.

## METHODS

### Study design and data samples

We drew country-by-country data on the availability of standard infection control practices in a number of LMIC’s health facilities from the Service Provision Assessment (SPA) surveys. These surveys, conducted by the Demographic and Health Survey (DHS) program with financial aid from the United States Agency for International Development (USAID), are publicly available, nationally representative, and de-identified cross-sectional surveys.

The DHS Program rigorously checks and cleans the data (i.e. all implausible data are removed) before making it publicly available to all researchers. Generally, all HCFs in each country are listed, and stratified random sampling is utilised to pick out a nationally representative sample of HCFs from the master list, including all public and private (including private for-profit and private-not-for-profit) HCFs. The SPA survey instrument includes indicators related to the WHO and USAID's methodology of Service Availability and Readiness Assessment (SARA). The SPA data and techniques can be used to produce accurate, reliable, and structured estimates of access to various types of health services (availability) and essential inputs for providing health services of high quality (readiness).

The initial implementation of the SPA questionnaire took place in 1997, and since then, a total of 30 surveys have been carried out across 17 different countries. There have been 16 surveys conducted in 8 countries since the survey instrument was revised in 2012.

We performed a secondary analysis of cross-sectional SPA survey data conducted after 2012, where the SPA-7 had been utilised. When more than one survey was done in the same country since 2012, we used the most current one except Senegal. Based on this criterion, eight LMICs (three sub-Saharan African, three Southeast Asian, and one Latin American and Caribbean) were involved in the analysis: Afghanistan (2018), Bangladesh (2017), the Democratic Republic of the Congo (DR Congo) (2017–18), Haiti (2017–18), Malawi (2013), Nepal (2015), Senegal (2016–17), and Tanzania (2015). The most recent Senegal SPA was not used in this study. In Senegal, a continuous SPA was conducted starting in 2012. The SPA sampling was designed so that sampling fractions were 20% for health posts and 50% for hospitals and health centres each year; as a result, we included two waves of data (SPA 2016 and 2017) to obtain a sample comparable to other nationally representative SPA surveys that include all hospitals and a fraction of lower-level facilities. The samples from Nepal, Senegal, and Tanzania from which we analysed data were regarded as nationally representative of their respective health systems. The assessments in Haiti, Malawi, and the DR Congo included all of the countries' health care facilities. The data from Bangladesh came from a sample of all public and private hospitals across the country. However, small private facilities were not included in the sample. The Afghanistan survey only looked at hospitals in urban areas of the seven main provinces. It is not representative of the country as a whole and cannot be compared directly to the other surveys. In this study, we used data from the facility inventory module of the SPA questionnaire.

### Measures

We assessed the availability of basic infection control items and PPE in health facilities in seven LMICs using data from recent SPA surveys. The availability of infection control items was evaluated using a list of six items (waste receptacle, sharps container, disinfectant, single-use disposable/auto-disable syringes, soap and running water or alcohol-based hand rub, and guidelines for standard precautions) according to the SARA reference manual [12] (**Table 1**). PPE includes four items as recommended by the WHO: gloves, medical masks, gowns, and eye protection [13]. We considered these items available in a facility if they were observed by the survey team in general outpatient areas or any service-specific area (i.e. delivery room). We calculated a score from zero to 6 for the number of infection control measures and zero to 4 for PPE.

**Table 1:** Definitions of items for basic infection control, and personal protective equipment

Domain	Items	Definitions
Basic infection control	Sharps container	Observed availability of a sharps container (safety box)
	Waste receptacle	Observed availability of waste receptacle (pedal bin) with lid and plastic bin liner
	Disinfectant	Observed availability of chlorine-based or other country-specific disinfectants used for environmental disinfection
	Single use – standard disposable or auto-disable syringes	Observed availability of single use syringes with needles (standard disposable or auto-disable)
	Soap and running water or alcohol based hand rub	Observed availability of piped water, water in bucket with specially fitted tap, or water in pour pitcher
	Guidelines for standard precautions	Observed availability of any guideline for infection control in health facilities
Personal protective equipment	Gloves	Observed availability of disposable latex gloves or similar non latex gloves
	Medical masks	Observed availability of flat or pleated surgical or procedure masks
	Gowns	Observed availability of non-sterile and fluid resistant gowns
	Eye protection (goggles or face shields)	Observed availability of goggles or face shields

Other variables of this study include facility type (hospital, health centres/clinic), managing authority (public, private), and location (urban, rural). Facility location type was not collected in the Nepal survey.

### Statistical analysis

In this study, we conducted the analysis in two stages: (1) univariate analysis – description of the item availability within each country with a 95% confidence interval (CI) – and (2) bivariate analysis – comparison of availability by facility type, managing authority, and location. For hospitals and all other facilities, we carried out separate descriptive analyses. We determined the average number of items for the hospitals surveyed in each study country and a similar average value for the health centers and clinics surveyed. We plotted the total number of items available by country. To compare the item availability by facility type, we plotted the average number of items for the surveyed private and public facilities – as well as the assessed rural and urban facilities – in each country. Stata 17.0 (StataCorp, College Station, TX, USA) was used for all data administration and analysis. We weighted all of our analyses using the sample weights included in the data set. To correct for the complex survey design, we utilised the ‘svyset’ command in Stata. All analyses are presented stratified by country.

## RESULTS

### Analytic sample

**Figure 1:**
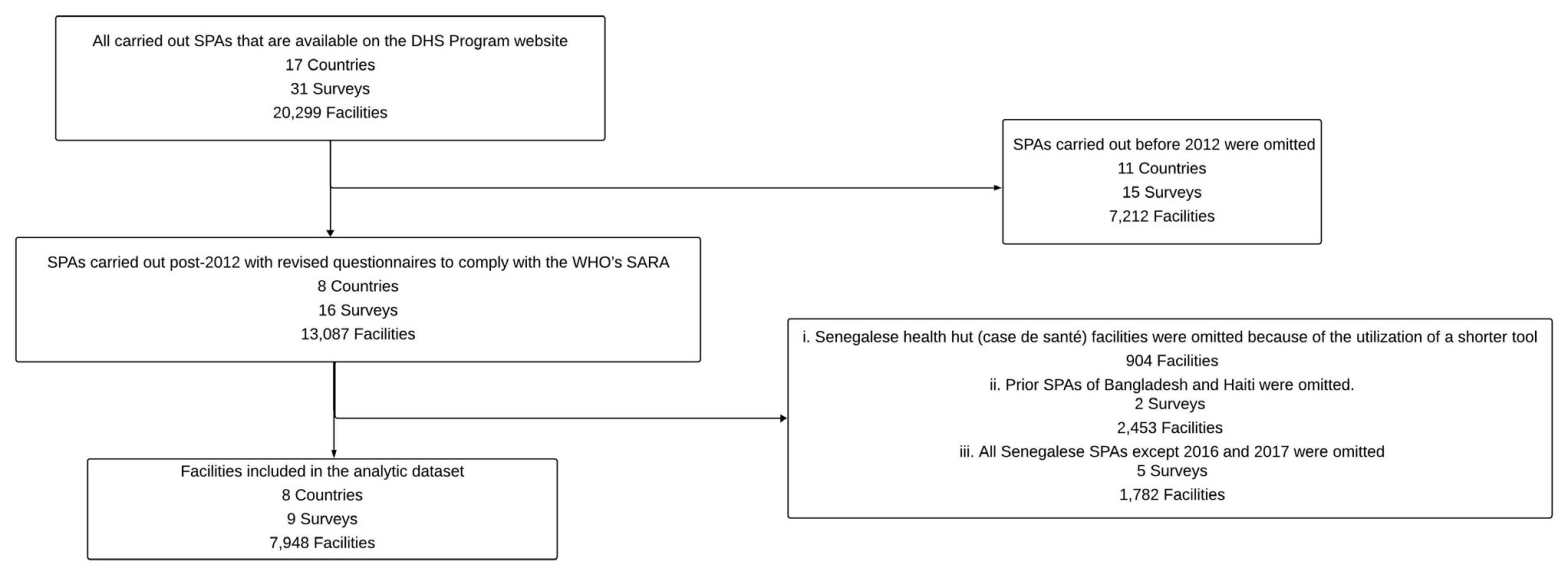
Schematic of Service Provision Assessment survey and creation of analytic data set.

After 2012, a total of 13 087 facilities across the eight countries, consisting of two low-income countries and six lower-middle-income countries, had data that was available (**Figure 1**). We merged the two surveys conducted in Senegal and only the most recent survey conducted in other countries, resulting in 8247 facilities for analysis. Among these, 7948 facilities, accounting for 96% of the total, were considered to have successfully completed the survey. Health hut facilities in Senegal (known as ‘cases de santé’), numbering 904, were excluded due to different selection methods. Response rates for these surveys are often high (Afghanistan: 89%, Bangladesh: 95%, DR Congo: 98%, Haiti: 97%, Malawi: 92%, Nepal: 97%, Tanzania: 99%), limiting the possibility of selection bias. In each country, the number of facilities sampled ranged from 142 in Afghanistan to 1524 in Bangladesh. Overall, 68.3% of the facilities that completed the survey were publicly funded, and 64.3% were located in rural areas (**Table 2**).

**Table 2:** Characteristics of eight countries in study sample, 2013–2018

Characteristics	Afghanistan	Bangladesh§	DR Congo	Haiti	Malawi	Nepal	Senegal‖	Tanzania	Overall
Geographic region	South Asia	South Asia	sub-Saharan Africa	Latin America and the Caribbean	sub-Saharan Africa	South Asia	sub-Saharan Africa	sub-Saharan Africa	
Income group	Lower middle	Lower middle	Low	Low	Low	Low	Low	Low	
Population (in millions)*†	37	161	77.3	10.7	16.4	28.2	15.1	51.8	
Population density (per km^2^)*	60	1237	34	389	174	197	79	59	
Urban population (% of total population)*†	26	34	42	59	16	18	44	31	
OOPS (% of the CHS)*†	79.3	73.9	41.6	39.9	6.7	59.4	51.2	25.8	
EHS (% of the CHS)*†	12.4	6.8	35.2	43.5	68.3	14.4	17.3	37.8	
Life expectancy at birth (years)*†	63	72.1	60.4	63.3	59.9	69.5	67.1	63.1	
COVID-19 deaths till May 2022†	7896	29131	1338	835	2640	11952	1966	803	
Data set information									
Year(s) of SPA data	2018	2017	2018	2017	2013	2017	2016- 2017	2015	
Field data collection timeline	November 2018 – January 2019	August 2017 – September 2017	October 2017 – April 2018	December 2017 – May 2018	June 2013 – February 2014	April 2015 – November 2015	March 2016 – November 2016, and March 2017 – December 2017	October 2014 – March 2015	
Number of facilities surveyed	142	1524	1380	1007	977	963	767	1188	
Sample or census	Sample	Sample	Sample	Census	Census	Sample	Sample	Sample	
Facility type									
Hospital	66 (46.30)	80 (5.28)	136 (9.82)	131 (12.98)	113 (11.53)	91 (9.49)	31 (4.03)	46 (3.9)	694 (8.73)
Health centres/clinic	76 (53.70)	1444 (94.72)	1244 (90.18)	876 (87.02)	864 (88.47)	872 (90.51)	736 (95.97)	1142 (96.1)	7254 (91.27)
Managing authority (ownership of facility)									
Public	23 (15.96)	1418 (93.02)			844 (61.19)	344 (34.12)	472 (48.34)	871 (90.42)	5427(68.28)
Private	119 (84.04)	106 (6.98)	536 (38.81)	663 (65.88)	507 (51.66)	92 (9.58)	169 (22.03)	331 (27.85)	2521 (31.72)
Facility location									
Urban	N/A	108 (7.09)	306 (22.2)	377 (37.49)	303 (31.01)	N/A	312 (40.62)	324 (27.27)	1872 (23.55)
Rural	N/A	1416 (92.91)	1074 (77.8)	630 (62.51)	674 (68.99)	N/A	455 (59.38)	864 (72.73)	5113 (64.34)

CHS – current health spending, EHS – external health spending, GDP – gross domestic product, OOPS – out-of-pocket spending, SPA – service provision assessment, USD – US dollar

*For nearest available year to service provision assessment year.

†Source: World Bank Global Development indicator [14,15].

‡Source: WHO Coronavirus (COVID-19) Dashboard [16].

§Excluding small private facilities, the data came from a sample considered representative of the country's health care system.

‖In Senegal, a continuous SPA was conducted starting in 2021. The SPA sampling is designed so that sampling fractions were 20% for health posts, 50% for hospitals and health centres each year; as a result, we included two waves of data to obtain a sample comparable to other nationally representative SPA surveys that include all hospitals and a fraction of lower level facilities [17]. A subsample of health huts (case de santé) was included in this survey. The approach used to pick health huts, on the other hand, was different, and their chances of being chosen were determined by the health posts with which they were linked. The current study does not include health huts. Data came from a sample considered representative of the country's health care system.

### Availability of infection control items and PPE across countries

Among the infection control items and PPE, overall, most surveyed facilities had high availability of single-use disposable/auto-disable syringes (90.9%, 95% CI = 89.9–91.8) and latex gloves (92.8%, 95% C.I = 92.0–93.7) (**Table 3**). Of infection control measures, guidelines for infection control were least available during the survey, with the lowest being 6.8% (95% CI = 4.9–9.3) in Nepal and the highest being 68.2% (95% CI = 65.2–71.1) in Malawi. Of the PPE items, eye protection was the least available during the survey, with the lowest being 5.4% (95% CI = 3.9–7.3) in Senegal and the highest being 28.2% (95% CI = 25.5–31.1) in Haiti. The availability of PPE and infection control items differs statistically across countries. Out of the facilities we analysed, only 1567 (19.71%) had all the infection control items, and 1023 (12.87%) had all the PPE needed for our total preventive measures calculations (**Table 4**).

**Table 3:** Percent of facilities with basic infection control items and personal protection equipment available across eight countries (n = 7948), 2013–2018

	Afghanistan		Bangladesh		DR Congo		Haiti		Malawi		Nepal		Senegal		Tanzania		All	
**Basic infection control items**	n	% availability (95% CI)	n	% availability (95% CI)	n	% availability (95% CI)	n	% availability (95% CI)	n	% availability (95% CI)	n	% availability (95% CI)	n	% availability (95% CI)	n	% availability (95% CI)	n	% availability (95% C.I)
Waste receptacle	132	92.7 (80.8–97.4)	533	35.0 (31.4–38.7)	234	17.0 (14.4–19.9)	448	44.4 (41.4–47.5)	648	66.3 (63.3–69.2)	122	12.7 (10.3–15.5)	641	83.6 (80.5–86.3)	841	70.8 (66.9–74.5)	3599	45.3 (43.8–46.7)
Sharps container	134	94.4 (81.2–98.5)	1024	67.2 (63.3–70.9)	1193	86.5 (83.4–89.1)	953	94.6 (93.1–95.9)	951	97.4 (96.1–98.2)	888	92.2 (89.6–94.1)	745	97.2 (94.6–98.5)	1166	98.2 (96.6–99.1)	7054	88.8 (87.6–89.8)
Disinfectant	134	94.6 (89.8–97.2)	613	40.2 (36.5–44.1)	1022	74.0 (70.5–77.2)	921	91.4 (89.5–93.1)	859	87.9 (85.7–89.7)	773	80.3 (76.2–83.8)	724	94.4 (92.1–96.1)	1046	88.0 (85.1–90.1)	6092	76.6 (75.2–78.0)
Single use – standard disposable or auto-disable syringes	126	88.4 (82.2–92.7)	1215	79.7 (76.2–82.9)	1259	91.2 (88.7–93.2)	969	96.2 (94.8–97.2)	948	97.1 (95.7–97.9)	928	96.4 (94.2–97.7)	649	84.6 (81.3–87.4)	1130	95.2 (93.1–96.6)	7224	90.9 (89.9–91.8)
Soap and running water or alcohol based hand rub	133	93.5 (81.2–97.9)	951	62.4 (58.4–66.2)	1061	76.9 (73.4–80.0)	877 1	87.1 (84.9–89.1)	825	84.4 (81.9–86.6)	636	66.1 (61.5–70.3)	764	99.6 (98.6–99.8)	1008	84.8 (81.5–87.7)	6255	78.7 (77.3–80.0)
Guidelines for standard precautions	38	26.5 (17.4–38.1)	290	19.0 (16.2–22.2)	458	33.2 (29.6–37.0)	407	40.5 (37.5–43.5)	666	68.2 (65.2–71.1)	66	6.8 (4.9–9.3)	458	59.7 (55.6–63.5)	483	40.6 (36.7–44.7)	2866	36.1 (34.7–37.4)
**Personal protection equipment**																		
Gloves	129	90.6 (75.5–96.8)	1225	80.4 (77.1–83.3)	1304	94.5 (92.4–96.1)	989	98.2 (97.2–98.9)	959	98.1 (97.1–98.8)	882	91.6 (88.6–93.9)	761	99.3 (97.4–99.8)	1133	95.4 (93.3–96.8)	7382	92.9 (92.0–93.7)
Medical masks	127	89.2 (78.8–94.9)	478	31.4 (27.9–34.9)	428	31.0 (27.7–34.5)	743	73.8 (70.9–76.4)	827	84.7 (82.2–86.8)	294	30.5 (26.9–34.4)	450	58.6 (54.6–62.5)	314	26.4 (23.1–30.1)	3661	46.1 (44.6–47.5)
Gown	90	63.7 (49.3–76.0)	465	30.5 (27.1–34.1)	1242	90.0 (87.5–92.1)	921	91.5 (89.6–93.1)	870	89.0 (86.8–90.9)	275	28.6 (25.1–32.3)	694	90.5 (87.4–92.9)	716	60.2 (56.1–64.3)	5273	66.3 (64.8–67.8)
Eye protection (goggles or face shields)	20	13.9 (9.1–20.6)	87	5.7 (4.4–7.3)	152	11.0 (8.9–13.5)	284	28.2 (25.5–31.1)	368	37.6 (34.7–40.7)	63	6.5 (5.1–8.5)	41	5.4 (3.9–7.3)	159	13.4 (11.0–16.2)	1174	14.8 (13.9–15.7)

CI – confidence interval

**Table 4:** Characteristics of health facilities by facility type in study sample, eight countries, 2013–2018

Characteristics	No. (%)			No. of facilities with relevant data available
	**All facilities (n = 7948)**	**Hospital (n = 694)**	**Health centres/clinics (n = 7254)**	
Managing authority				7948
*Public*	5427 (68.28)	238 (34.29)	5189 (71.53)	
*Private*	2521 (31.72)	456 (65.71)	2065 (28.47)	
Facility location				6985
*Urban*	1872 (23.55)	397 (57.21)	1475 (20.33)	
*Rural*	5113 (64.34)	205 (29.54)	4908 (67.66)	
Quality assurance activities				7870
*Performed*	3258 (40.99)	479 (69.02)	2779 (38.31)	
*Not performed*	4612 (58.03)	206 (29.68)	4406 (60.74)	
Routine user fee or charges for client service				
*Fixed for all services*	2299 (28.92)	119 (17.15)	2180 (30.05)	
*Separate for each service*	3293 (41.44)	504 (72.62)	2789 (38.45)	
*No routine user fee*	2356 (29.64)	70 (10.09)	2286 (31.51)	
External supervisory visit to facility				7830
*Received, within the past 6 months*	6549 (82.40)	535 (77.08)	6014 (82.91)	
*Received, more than 6 months ago*	834 (10.49)	97 (13.98)	737 (10.16)	
*Not received*	448 (5.64)	53 (7.64)	395 (5.45)	
Facility had all items				
*For basic infection control*	1567 (19.71)	274 (39.48)	1293 (17.82)	
*For personal protection equipment*	1023 (12.87)	296 (42.65)	727 (10.02)	

### Health facility characteristics by facility type

Of the 7948 facilities included in our analysis, 694 were hospitals. Compared with hospitals, health centres and clinics were less likely to be privately owned (28.47 vs. 65.71%), located in urban areas (20.33 vs. 57.21%), perform quality assurance activities (38.31 vs. 69.2%), and receive distinct user fees or charges for every client service (38.45 vs. 72.62%) (**Table 4**). Over 75% (535 of 694) of the hospitals received an external supervisory visit to the facility within the past six months of the survey, compared with about 80% (6014 of 7254) of the health centres and clinics. A low percentage of investigated facilities possessed all elements in either of the two domains. About 40% (274/694) of hospitals and 18% (1293/7254) of health centres and clinics looked to have all the essential infection control materials. Regarding PPE, the equivalent numbers were about 43% and 10%, respectively.

### Availability of all infection control items and PPE by facility type

**Figure 2:**
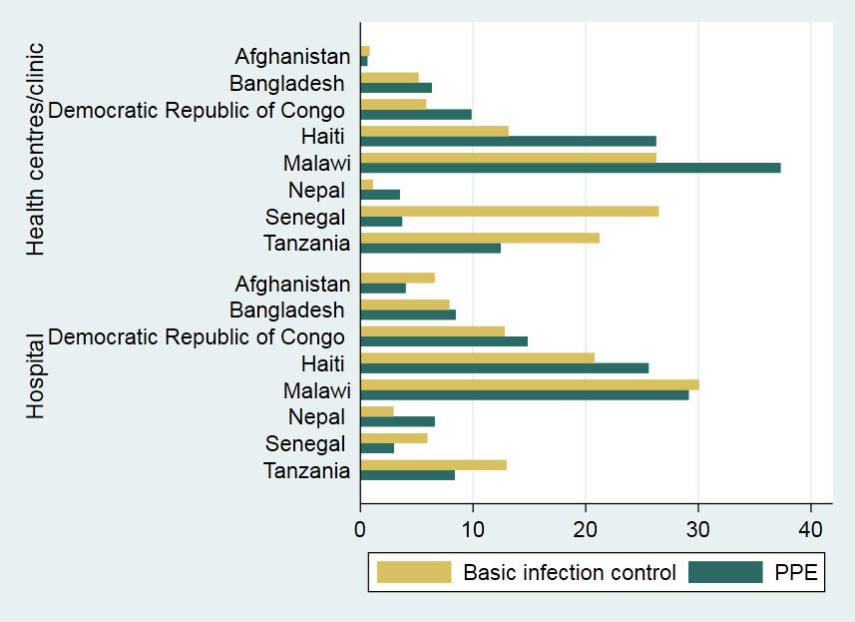
Availability of all six infection control items and four personal protective equipment by facility type, eight countries, 2013–2018.

At the national level, the availability of all items for basic infection control in health centres/clinics ranged from 1.11% in Nepal to 26.48% in Tanzania and from 2.93% in Nepal to 30.00% in Malawi for hospitals (**Figure 2**). The availability of all four PPE items at health centres/clinics ranged from 3.51% in Nepal to 37.30% in Malawi, while the availability in hospitals ranged from 2.98% in Senegal to 29.14% in Malawi.

### Inequalities in the availability of infection control items and PPE by managing authority

**Figure 3:**
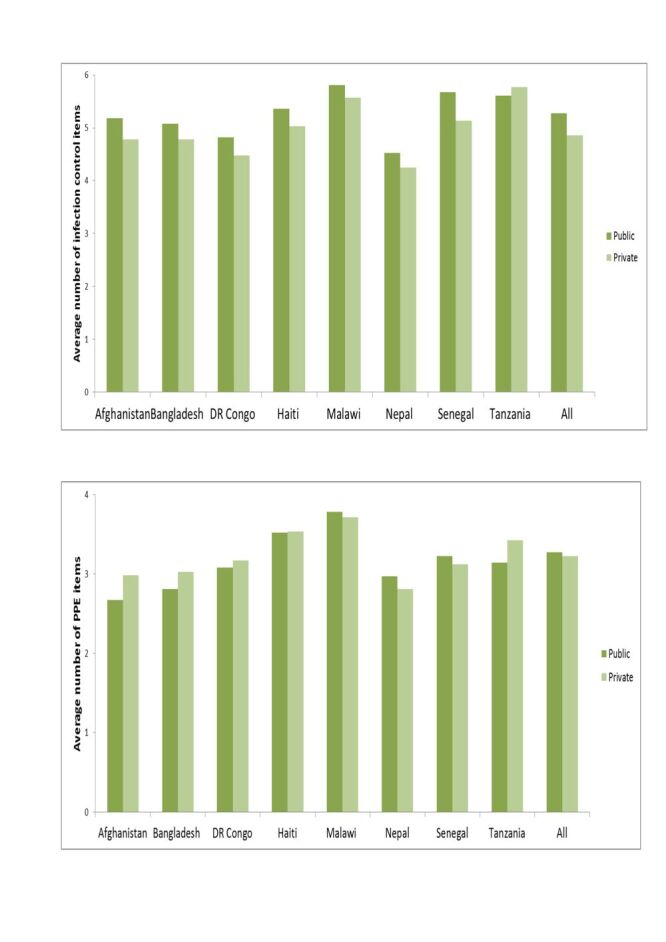
Differences in the availability of infection control items and personal protective equipment in hospitals by managing authority, eight countries, 2013–2018.

Regarding infection control items, the average values for the public hospitals were higher than the private hospitals in Tanzania but lower in Bangladesh, the DR Congo, Haiti, Malawi, Nepal, and Senegal (**Figure 3**). PPE was easier to find in public hospitals in Malawi, Nepal, and Senegal compared to private hospitals.

**Figure 4:**
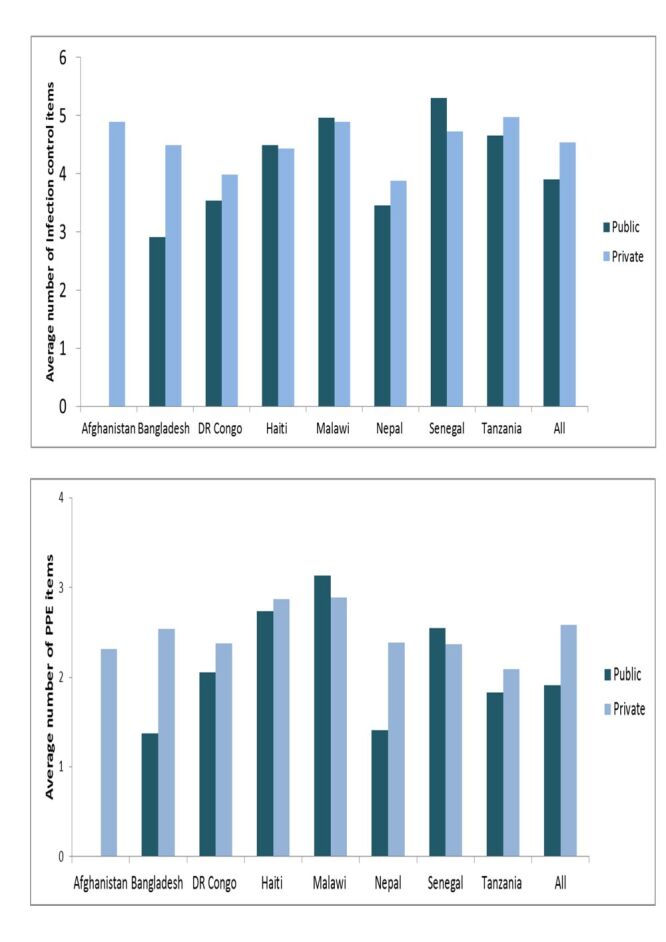
Differences in the availability of infection control items and personal protective equipment in health centres/clinics by managing authority, eight countries, 2013–2018.

The availability of infection control items and PPE in private health centres and clinics was significantly lower in Malawi, and Senegal compared to those in public health centres/clinics and higher in Bangladesh, the DR Congo, Nepal, and Tanzania (**Figure 4**).

### Inequalities in the availability of infection control items and PPE by facility location

**Figure 5:**
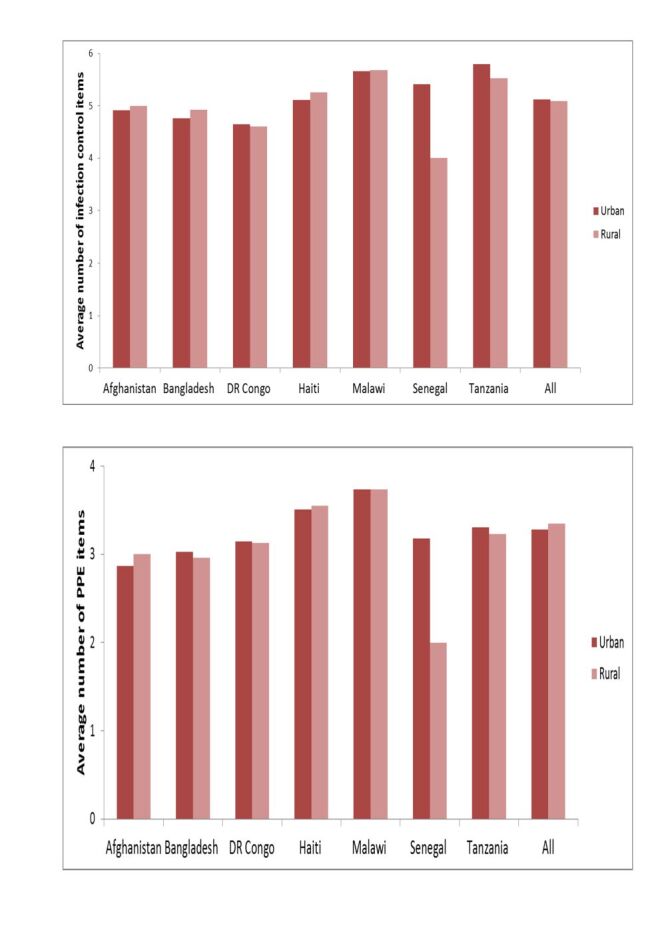
Differences in the availability of infection control items and personal protective equipment in hospitals by location, seven countries, 2013–2018.

Urban hospitals in the DR Congo, Senegal, and Tanzania had more available infection control items than rural hospitals, and urban hospitals in Bangladesh, and Haiti had fewer items (**Figure 5**). Regarding PPE, urban hospitals in Bangladesh, the DR Congo, Senegal, and Tanzania had higher quantities than rural hospitals. Malawian urban hospitals had a similar amount of PPE as rural hospitals.

**Figure 6:**
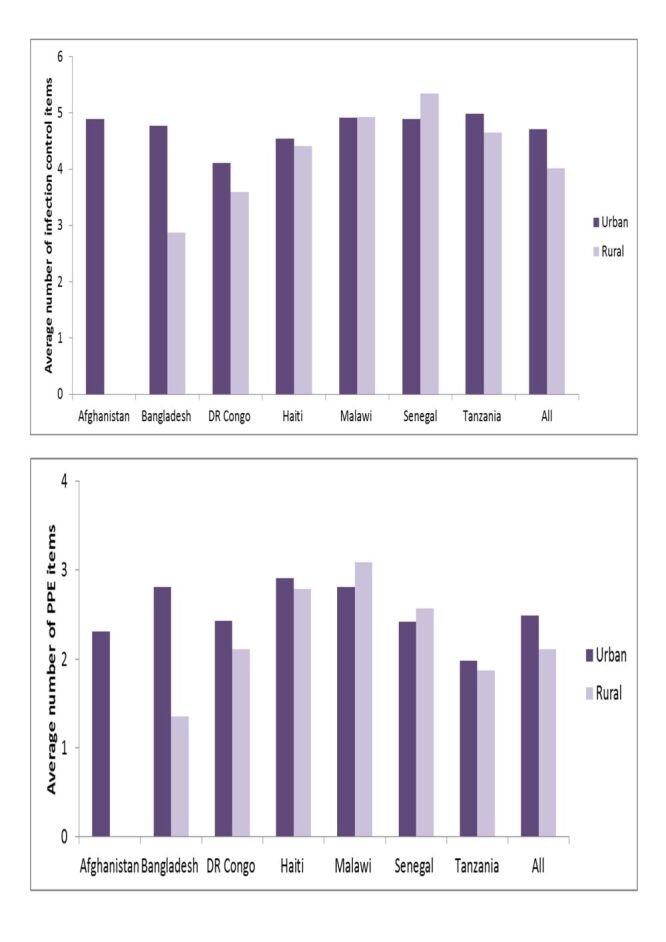
Differences in the availability of infection control items and personal protective equipment in health centres/clinics by location, seven countries, 2013–2018.

Additionally, urban health centres in Bangladesh, the DR Congo, Haiti, and Tanzania exhibited had more infection control items and PPE than their rural counterparts (**Figure 6**). However, this improvement was not observed in Malawi or Senegal.

## DISCUSSION

Our cross-country analysis of the infection control items and PPE availability in 7948 health facilities indicated substantial and extensive inadequacies in the availability of those items. This work updates and builds on previous comparative assessments of basic infection control items and PPE and identifies potential for health infrastructure improvement. About two out of every five hospitals had all the PPE and necessary items for infection control. About one out of ten health centres or clinics had all PPE, and about one out of five had infection control items. Overall, about 80% of facilities in all the study countries don't have all the elements needed to control infections, and 87% of them don't have adequate PPE measures. Among all the measures, gaps were particularly apparent in guidelines for infection control and eye protection. Single-use auto-disposable syringes and latex gloves were more common across the countries. Most health facilities in the countries studied have a scarcity of infection control items and PPE. An estimated 56% of facilities do not have waste receptacles, 64% do not have infection control guidelines, and 85% do not have eye protection.

Although the average number of items varied across countries in the study, the disparity between facilities within the same country was much more significant. While all of the countries in the study seemed to be capable of equipping some facilities adequately, they all failed to ensure the consistent availability of infection control items and PPE items in their entire health systems.

In Bangladesh, the availability of almost all items was less than 80%. Bangladesh also showed the widest gaps in coverage across subgroups between public and private health centres and between urban and rural health centers. These results are in line with another multi-country study that examined the capability of health facilities in Bangladesh, Haiti, Malawi, Senegal, and Tanzania to offer newborn care [18]. Even after successfully handling the Ebola outbreak, the DR Congo continues to face challenges with a low percentage of infection control measures and available PPE items. Haiti has the fewest gaps in the availability of infection control items between public and private health centres, consistent with the findings from another study [19]. Nearly all of the materials were available in Malawi. The public hospitals and health centres/clinics in Senegal had more infection control items and PPE than the private hospitals and health centers. It fits with the results of another study, which found that efforts were made to increase core health services in the public sector in line with Senegal's national referral policy [20,21].

In many countries, publicly owned health centres and clinics located in rural areas performed poorly, although not in all. According to research conducted in various countries, in certain countries, the public sector provides better care than the private sector, while the public sector provides worse care in other countries [22–24]. Our analyses, which used a standard measure and nationally representative data, not only yielded a comparable result but also indicated that, within any particular country, the public sector’s tendency to provide care of higher – or lower – quality than the private sector might vary depending on facility type. Our findings confirm the findings of prior single-country studies by highlighting the significant challenges in providing adequate care in rural areas due to severe shortages in the availability of IPC measures In LMICs, rural HCF represents a large proportion of the health care providers in health systems [11], and the majority of the population still lives in rural areas [25]. The incommensurate shortage of standard precaution items in rural areas has consequences for infectious diseases’ prevention and control and quick responses to COVID-19.

Several studies found that hospitals had consistently greater availability of health services in comparison to all other facility types [10,11,26]. In a similar way, our study showed that there is a difference in the availability of infection control items and PPE between hospitals and dispensaries/clinics in all countries. Hospitals serve a larger number of patients than all other facility types and provide a wider range of services; hence, it is likely that additional resources will be invested in hospitals. Hospitals may have been prioritised for PPE and IPC due to the type of care provided (i.e. length of stay, wounds, invasive procedures, and surgeries), posing a risk for HAI. But only if we pay more attention to the supplies and infrastructure at the health centres and clinics that serve as the population’s first line of care could this type of care be a solid platform in low-income countries for addressing a variety of health concerns like respiratory contagion (COVID-19).

Many health care systems lack basic infection prevention items and PPE. The findings highlight an urgent concern: approximately one in every five facilities in all studied countries has basic items for infection control, and one in every eight has adequate PPE measures. These findings reveal a significant and concerning discrepancy in HCFs real readiness for IPC measures across the board, highlighting a crucial need for prompt attention and legislative intervention. These disparities are visible not only when comparing various countries but also, perhaps more importantly, inside the same country. While our data show within-country inequalities, the overall deficits were far more pronounced. The availability of PPE and fundamental shortcomings in infection control supplies affect the entire health system infrastructure, not just regional differences.

In order to overcome these shortcomings and bridge the disparity in preparedness, it is crucial to enact the recommendations for improving logistical systems put forth by established resources such as the Lancet Global Health Commission on High Quality Health Systems (HQSS Commission) [27]. Their recommendations provide a solid framework for increasing the availability and distribution of necessary commodities. By incorporating these established recommendations, we may contribute to the improvement of policies aimed at enhancing performance at both the national and subnational levels. It is particularly relevant in the context of logistics systems, which play a crucial role in enabling the timely monitoring of essential resources. Consequently, ensuring adequate equipment for health care facilities allows them to effectively manage infectious diseases and safeguard the well-being of HCWs and patients.

### Strengths and limitations

This study has various strengths. First, the present study provides information on basic infection control items and PPE availability and compares them across key strata within countries. Second, this study identified gaps in the standard precaution items availability in health facilities by facility type (hospital, health centres/clinic), managing authority (public, private), and location (rural, urban). Third, a comparison of the item availability in almost 8000 health facilities across countries revealed insufficient availability of infection control and PPE items, which represent substantial health risks in health facilities and would influence improved decision-making and protection of health. Finally, the findings of this study are essential for enhancing facility conditions to meet standard services, raising accountability, and improving evidence-based strategies and effective programmes. These findings guide the allocation of resources to facilities lacking adequate services, ensuring their efficient utilisation.

There are several notable limitations to this research. First, the study utilises health facility data from specific countries in Southeast Asia, sub-Saharan Africa, Latin America, and the Caribbean. Understanding the extent of insufficient availability, especially in Southeast Asia with its substantial low- and middle-income population, is essential. Second, the analysis is confined to SPA data from only eight LMICs, making it challenging to generalise the findings to other countries or regions. Third, our estimates might be outdated due to varying data sources and collection times among countries, not accurately reflecting the current facility situations, given common issues like periodic supply interruptions and stock-outs in the region [25,28]. Fourthly, the study lacks recent data from all countries, limiting the inclusion of comprehensive SPA data. More frequent facility evaluations, like SPA and SARA, are necessary to gain a better understanding of health facility status. Including more countries would enhance the capability to assess how national factors contribute to facility availability. Coordinating data collection methods and tools would enable monitoring and evaluation of improvements at national, international, and regional levels. Fifthly, the study's measures are conservative; we only require these items to be available in any part of the facility, not necessarily every part. Finally, while the research examines disparities by facility type, managing authority, and location, it does not investigate other critical factors, such as different populations' access to health facilities. Additionally, although the study provides a general overview of availability variations based on managing authority and location, it's important to note that facilities within the same location or under the same authority can differ significantly.

## CONCLUSIONS

This study indicates the extent and complexity of the inadequacy and deficits in the most fundamental standard precaution items to avert infection for the safety of patients and HCWs. Reducing deficits and enhancing efficiency in the conversion of health-system inputs into facilities' availability of standard precaution items for infection control – and, ultimately, the conversion of facility preparedness to effectively combat infectious diseases like Ebola and COVID-19 – are critical milestones on the road to both accessible and efficient health care. More generally, as a pragmatic and successful solution, the existing deficits must be addressed by implementing more agile monitoring mechanisms similar to logistics systems. Integrating these advanced monitoring systems would provide quick alerts of existing shortages to subnational and national officials, allowing for prompt responses.
